# Combination Therapy for Hypothyroidism: Rationale, Therapeutic Goals, and Design

**DOI:** 10.3389/fendo.2020.00371

**Published:** 2020-07-08

**Authors:** Ritu Madan, Francesco S. Celi

**Affiliations:** Division of Endocrinology, Diabetes and Metabolism, Virginia Commonwealth University, Richmond, VA, United States

**Keywords:** hypothyroidism, liothyronine, levothyroxine, combination therapy, pharmacokinetics

## Abstract

Hypothyroidism is a common condition with a wide spectrum of etiologies and clinical manifestations. While the majority of patients affected by hypothyroidism respond well to levothyroxine, some patients do not and complain of symptoms despite adequate replacement. There is evidence in experimental models of hypothyroidism that levothyroxine alone may not be able to deliver an adequate amount of T3 to all the tissues targeted by the hormonal action, while liothyronine/levothyroxine combination therapy can. The results of clinical studies directed to assess the effectiveness of liothyronine/levothyroxine combination therapy on the amelioration of hypothyroid symptoms have been disappointing. Most of the trials have been short and underpowered, with several shortcomings in the study design. There is consensus that an adequately powered clinical trial should be developed to prove or disprove the efficacy and effectiveness of therapies other than LT4 alone for the treatment of hypothyroidism, and to assess which group of patients would benefit from them. Here we present some considerations on the technical aspects and necessary tradeoffs in designing such a study with a particular focus on study population selection, choice of endpoints, and study drugs formulation and regimen.

## Introduction

The majority of the patients affected by hypothyroidism are successfully treated with levothyroxine (LT4), which is considered by U.S. professional organizations to be the standard of care for this condition (1, 2). Unfortunately, a sizable minority of patients, up to 40%, still complains of hypothyroid symptoms despite achieving the target TSH (3, 4), resulting in repeated dose adjustments, additional testing, patient dissatisfaction, and provider frustration. Obvious advantages of LT4 thyroid hormone replacement therapy include well-known pharmacokinetics characteristics which allow for daily administration, its safety profile, and the wide array of dose strengths which allow for precise titration. Moreover, the use of TSH as proxy of euthyroidism provides a reliable therapeutic target for dose adjustments. LT4 therapy is based on the assumption that the peripheral conversion of exogenous T4 is able to provide adequate supply of T3 to the various targets of the hormonal action, and that a state of pituitary euthyroidism (as indicated by a TSH within the normal range) equates to a state of generalized euthyroidism. Of interest, while LT4 therapy is extremely effective in normalizing TSH, it does not appear to completely normalize the serum concentrations of T4 and T3, since the ratio T4:T3 is skewed toward an increase of T4, while T3 levels are normal to low (5). The clinical significance of these changes in thyroid hormone is not clear, but it may reflect a state of relative imbalance in the thyroid hormone axis homeostasis, which in turn could cause the persistence of hypothyroid symptoms in some individuals.

The addition of liothyronine (synthetic T3, LT3) to LT4 in different LT3:LT4 combination therapy schemes, or the use of desiccated thyroid extracts (DTE), are attempts to replicate the endogenous production of thyroid hormone. The scientific rationale for these treatments is based on the landmark experiments of Morreale d'Escobar in an elegant experimental model of hypothyroidism, which demonstrated that LT4 alone is not sufficient to reverse hypothyroidism in all tissues (6), and only administration of a combination of LT3 and LT4 could restore normal tissue content of T3 (7). Contrary to rodents, in humans the majority of the thyroid gland production is T4, which is then converted to T3 in the peripheral tissues. By mathematical modeling, Pilo and colleagues have estimated that the daily release of T3 from the thyroid gland is approximately 3.3 mcg ^*^ m^−2^, while the peripheral conversion of T4 account for another 12.7 mcg ^*^ m^−2^ (8). These data have provided the theoretical underpinnings to design therapeutic regimens aimed to restore the “physiologic” concentrations of thyroid hormone.

Several clinical trials (Table 1) have been conducted with various treatment schemes, duration, and endpoints (9–16, 18–24). The results of these studies have been inconsistent, and in the aggregate LT3:LT4 combination therapy or DTE (25) have not shown superiority to LT4 in relieving symptoms of hypothyroidism. Nonetheless, some patients treated with combination therapy or DTE showed remarkable improvement in some of their symptoms, raising the question of how to identify which subgroup of patients could benefit from such treatment.

**Table 1:** LT3:LT4 combination therapy studies for the treatment of hypothyroidism.

**References**	**Study design**	**Patient No**.	**Type of Hypothyroidism**	**Drug formulation**	1*Outcome	**Other outcomes**
Bunevicius et al. (9)	Randomized, blinded crossover with two 5-wk periods	26	Autoimmune + postsurgical (11+15)	LT4 at usual dose or minus 50 mcg and adding LT3 at 12.5 mcg with LT4:LT3 ratio 3:1 to 15:1	•Improvement in mood on POMS scale in thyroid cancer patients on combination •Improvement in digital symbol test and visual scan test in thyroid cancer patients on combination therapy	•No change in Beck depression inventory test and Spielberger State-Trait Anxiety Inventory (SSTAI) •serum cholesterol similar in both groups •SHBG and pulse rate higher in combination treatment group
Bunevicius et al. (10)	Randomized, blinded crossover with two 5-wk periods	10	Postsurgical, subtotal thyroidectomy for Graves' disease	LT4 at usual dose or minus 50 mcg and adding LT3 at 10 mcg with LT4:LT3 ratio 5:1 to 10:1	No statistically significant difference in mood, cognitive Scale and hypothyroidism symptoms score	6 patients preferred combination therapy, 2 patients preferred monotherapy and 2 had no preference
Sawka et al. (11)	Randomized, blinded controlled, 15 week	40	Autoimmune	20 patients LT4 only and 20 LT4+T3 (Pre-study LT4 dose reduced to 50% and LT3 added 12.5 mcg twice daily	No statistically significant difference in symptoms, mood, depression scores or general well-being scores	
Clyde et al. (12)	Randomized, double blind, placebo controlled,4 months trial	44	Autoimmune + postablative + postsurgical +post EBRT (39+10+2+1)	LT4 monotherapy usual dose (13) vs. reduced dose of LT4 (usual-50 mcg)+LT37.5 mcg twice daily. Doses adjusted every 5 weeks	•No difference in TSH at 4 months •No differences in QOL assessment between treatment,1/13 neuro cognitive assessment significantly different in favor of monotherapy	
Walsh et al. (14)	Randomized, blinded controlled, 2-group crossover with two 10-wk periods, separated by 4 week of T4 alone	110	Autoimmune+ postablative + postsurgical (94+4+12)	LT4 at usual dose followed by LT4+LT3 (*n =* 56). Group 2 had reverse order (*n =* 54). For combined treatment, L4 usual dose minus 50 mcg and adding LT3 at 10 mcg	•No significant difference in quality of life score •Higher GHQ28 score indicating worse psychological well-being in combination group •No difference in cognitive scores	No difference in treatment satisfaction scores
Siegmund et al. (15)	Randomized, blinded crossover with two 12-wk periods	23	Postsurgical + autoimmine (21+2)	LT4 at same dose or 95% LT4 with 5% substituted as LT3 equivalent to an absorbed molar mixture of 14:1. After 6 weeks, dose was adjusted	•TSH significantly lower in the combination therapy •No significant change in mood, cognition and general well-being scores	1 person had atrial fibrillation on combination with suppressed TSH
Appelhof et al. (16)	Randomized, controlled 15 week	130	Autoimmune	LT4 alone (17) vs. LT4:LT3 (*n =* 46) 10:1 vs. LT4:LT3 (*n =* 47) 5:1, combination adjusted at 5 weeks	Patient preferred combination therapy. Preference for treatment as –LT4 alone 25%, LT4:LT3 10:1 41%, LT4:LT3 5:1 42%	•TSH levels lower in patients receiving combination •Significant weight loss in LT4:LT3 5:1 group (1.7 kg) •No difference in mood, and in general well-being scores
Escobar-Morreale et al. (18)	Randomized, double blind, crossover design with three 8-wk periods	26	Autoimmune + postablation for Graves or MNG (23+5)	•14 patients received LT4 100 mcg alone for 8 week, 13 patients then LT4 75MCG+LT3 5 mcg for 8 weeks, followed by LT4 87.5 mcg+LT3 7.5 mcg (*n =* 12) •14 patients received LT4 75MCG+LT3 5 mcg for 8 weeks, followed by LT4 100 mcg 8 weeks, followed by LT4 87.5 mcg+LT3 7.5 mcg for 8 weeks	•No difference in LT4 and LT4+LT3 75+5 mcg group in POMS, on the Digit Symbol Substitution Test, or on the Visual Scanning Test. Slight improvement in the backward and total scores of the Digit Span Test •No difference between the LT4+LT3 87.5+7.5 mcg group and previous treatment in terms of POMS or the Digit Span Test. Better performance Digit Symbol Substitution Test and the visual scanning test	12 patients preferred LT4+LT3 75+5 mcg, 2 preferred LT4, 6 preferred LT4+LT3 87.5+7.5 mcg, 6 had no preference
Rodriguez et al. (19)	Randomized, blinded crossover with two 6-wk periods	27	Autoimmune+ postablative + postsurgical (23+4+3)	LT4 at usual dose or minus 50 mcg and adding LT3 at 10 mcg with LT4:LT3 ratio 5:1	No difference in fatigue score between groups	•No difference in depression score, hypothyroid symptoms and TSH •7 preferred LT4, 12 preferred LT4 +LT3, 8 had no preference
Saravanan et al. (20)	randomized, parallel group, controlled 12 months trial	697 (573 analyzed)	•Not mentioned •Excluded thyroid cancer patients	LT4 at usual dose (*n =* 353) or minus 50 mcg and adding LT3 at 10 mcg(*n =* 344)	Improvements in GHQ caseness at 3 months but not GHQ Likert scores and the initial differences were lost at 12 months	Improvements in GHQ Hospital Anxiety and Depression questionnaire-anxiety scores at 3 months but Hospital Anxiety and Depression questionnaire-depression, thyroid symptoms, or visual analog scales of mood and the initial differences were lost at 12 months
Valizadeh et al. (21)	randomized, double blind, parallel group, 4 months trial	71	Autoimmune + postablative +postsurgical (46+12+2)	LT4 at usual dose (*n =* 35) or minus 50 mcg and adding LT3 at 6.25 mcg BID (*n =* 34). Typical LT4:LT3 4:1,LT4 doses adjusted after 1 month to normalize TSH	The overall score of GHQ-28 was not significantly different between LT4 and combined LT4/LT3 groups. Of the four subscales of the GHQ-28, the only significant difference was observed in the mean score of anxiety/insomnia. In favor of combined LT4+LT3 group	
Nygaard et al. (22)	Randomized, blinded crossover with two 12-wk periods	59	Autoimmune	LT4 at usual dose or minus 50 mcg and adding LT3 at 20 mcg with mean LT4:LT3 ratio 4:1.dose of LT4 adjusted every 4 weeks	•Significant beneficial effect on QOL and depression score (7/11 measures) in favor of combination therapy •Significant beneficial placebo effect with t4 therapy in 10/11 measures	49% preferred combination, 15% preferred LT4, 35% had no preference
Fadeyev et al. (23)	Randomized, controlled, non-blinded 6 month study	36	•Not mentioned •Newly diagnosed patients	LT4 at dose 1.6 mcg/kg (*n =* 20) vs. LT4 dose 166 mcg/kg-25 mcg +LT3 12.5 mcg daily	•No difference in TSH •Total and LDL cholesterol significantly lower in combination group	No difference in preference for treatment for either regimen
Kaminski et al. (13)	Randomized, blinded crossover with two 8-wk periods	32	Autoimmune + postablative + postsurgical (23+3+6)	•Patients were on stable dose of 125 or 150 mcg LT4 before entering study •LT4 at usual dose or LT4 75 mcg+LT3 15 mcg with LT4:LT3 ratio 5:1	•Free t4 levels were significantly lower and resting HR slightly higher with combination vs. monotherapy •No changes observed in QOL questionnaire, lipids, BMI	
Krysiak et al. (24)	Quasiblind, randomized	39	Post-hemithyroidectomy, females only with symptoms of hypothyroidism	Usual levothyroxine dose vs. LT4/LT3 combination in ratio 5:1	•Combination therapy had beneficial effect on 2/6 domains in female sexual function index (FSFI) •No difference in depression score between treatment groups	No difference in TSH between groups.

The need and design of additional trials aimed to assess the efficacy and effectiveness of LT3:LT4 combination therapies or DTE in the treatment of patients with hypothyroidism is being actively debated among researchers and practitioners. On the one hand, the inconsistency of the data obtained in the previous trials tends to dissuade from embarking on what would be another negative study; on the other hand, one could argue that a rigorous study design based on rational decisions on study population, outcomes, and drug formulation could be able to address an unresolved prevalent and disabling clinical problem.

Depending on the priorities of the individual investigator/practitioner target population, drug formulation and administration as well observation duration would vary significantly. These differences should not be a concern as long the various tradeoffs in the decision-making process are well-identified and addressed in a manner that is consistent to the study question/therapeutic goal. Conversely, study design or treatment scheme decisions based on extraneous constrain would inevitably result in an inadequate intervention. The goal of this manuscript is to discuss these variables in the context to provide the rationale to design future LT3:LT4 combination therapy clinical trials.

## Target Population

### Etiology

Hypothyroidism is a highly prevalent condition (26, 27), but extremely heterogeneous in terms of severity (i.e., degree of endogenous production of thyroid hormone loss), etiology, and comorbidities. In the USA, autoimmune thyroid disease is by far the most common cause of hypothyroidism (frequently detected in the subclinical state), followed by radioiodine ablation for Graves' disease and thyroidectomy. Currently, the vast majority of the prescriptions for LT4 are directed to patients that are affected by subclinical hypothyroidism, which by definition is characterized by residual production of thyroid hormone sufficient to maintain the circulating levels within the normal range. Conversely, individuals who have undergone total thyroidectomy and remnant ablation have no residual endogenous production of thyroid hormone. Clearly, the pathophysiology of the thyroid axis of these two conditions at the extremes of the spectrum of hypothyroidism differs immensely, and it should be taken in consideration when designing a study.

Two diametrically opposed approaches to recruiting the study population have both benefits and drawbacks, which should be carefully evaluated in designing and powering a trial. A recruitment strategy that includes self-described “dissatisfied patients” or “all comers” would be representative of the statistical universe of patients affected by hypothyroidism, and provide an agnostic approach to the question of who could benefit the most from non LT4-only treatment. To achieve statistical power to detect significant differences, such a study would require a population comparable in size (thousands) to the ones performed for diabetes or cardiovascular disease. An important caveat inherent to this approach is that the clinical significance of the study endpoint (*see below*) may not be clearly appreciated by practitioners or lay public. A specific consideration on the strategy of recruiting all comers lay in the diagnosis of hypothyroidism since the sole treatment with thyroid hormone replacement therapy does not constitute a clinical diagnosis, and patients may not recollect the initial TSH level that led to the initiation of treatment. Corrective actions to improve the selection process could include a screening process that takes into account previous documentation of clearly pathologic levels of TSH, or evidence of treatment (surgery or radioactive iodine). In the absence of such evidence, one could use as proxy the presence of anti-TPO antibodies, although this would not equate to lack of endogenous production of thyroid hormone. One could propose to confirm the diagnosis of hypothyroidism by assessing the rise in TSH following discontinuation of the therapy, but this extra step would cause ethical concerns and likely decrease the ability of recruiting study patients.

A conceptually opposite recruitment strategy to the “all comers” consists of developing very strict inclusion criteria, which would provide the best odds to achieve a statistical and clinically relevant outcome by increasing the signal to noise ratio. The specular drawback of this decision would be the risk of excluding patients who may benefit from non-LT4-based therapy since at the present time, the understanding of the “ideal” target population is based on limited observations, or inferred from the pathophysiology of the various forms of hypothyroidism.

### Symptomatology

If one would approach the study question from a patient-centered perspective, the primary driver of the decision of switching from LT4 to LT3:LT4 combination or DTE would be purely based on symptoms. This is certainly a valid question, but of extreme complexity from the technical perspective. In fact, setting aside the diagnosis of hypothyroidism and its severity (*discussed above*), researchers are faced with the heterogeneity of the manifestations of hypothyroidism, which are a panoply of aspecific signs and symptoms that have significant overlap with non-affected patients and with patients affected by other conditions (28). Importantly, very often Hashimoto's thyroiditis disease is associated with other autoimmune conditions that are also associated with aspecific symptoms, creating a significant confounder in the attribution of the symptoms to hypothyroidism. Another relevant confounder is represented by the slow and progressive onset of thyroid failure, which makes it extremely difficult from the patients' perspective to attribute with certainty symptoms to hypothyroidism rather than to other co-morbidities or to the aging process. Moreover, given the chronicity of this condition, it is challenging to discern from the patient's perspective the difference of the state of health prior to the development of hypothyroidism from an aspirational state of well-being. All these confounders play a critical role in selecting the study outcome and measures, which will be addressed in details in the next section.

By limiting the study population to specific causes of hypothyroidism, one could significantly reduce the heterogeneity of the sample population, increasing the power of the study to detect statistically significant differences in the primary outcome of the study. To this end, the most appealing strategy would be to limit the recruitment to thyroidectomized patients. The two major advantages of this choice reside in the fact that patients who have undergone total thyroidectomy have by definition no residual thyroid hormone production, and transition acutely from a state of euthyroidism to being entirely dependent from exogenous thyroid hormone therapy. This approach would work well if the goal of the study resides in assessing laboratory-based endpoints. On the other hand, while patients who have undergone total thyroidectomy represent an ideal “experimental platform” to study the effects of thyroid hormone replacement therapy, they are a small minority of patients affected by hypothyroidism, and thus are not representative of the condition.

### Genetic Polymorphisms

Genetic background plays an important role in the response to drugs, and several genetic polymorphisms in the thyroid hormone metabolism and signaling (Table 2) have been associated with changes in thyroid hormone levels and to some degree with response to therapy (29–35).

**Table 2:** Common genetic polymorphisms (Single Nucleotide Polymorphisms-SNP) associated with thyroid hormone axis and response to therapy.

**References**	**Polymorphism(s)**	**Gene**	**Function**	**Finding**	**Notes**
Mentuccia et al. (29) Panicker et al. (32)	Thr92Ala rs225014	Type 2 deiodinase (DIO2)	T4 → T3 conversion	Decreased activity, associated with improved response to LT3:LT4 therapy	Synergistic effects with other polymorphisms
Peeters et al. (30)	Asp727Glu rs1991517	TSH receptor (TSH-R)	TSH receptor	Lower TSH levels, no changes in thyroid hormone	
Peeters et al. (30)	C785T rs11206244	Type 1 deiodinase (DIO 1)	rT3 → T2 T4 → T3 conversion	Correlation between the T allele and rT3 levels	Interpreted as loss of function
Peeters et al. (30)	A1814G rs12095080	Type 1 deiodinase (DIO1)	rT3 → T2 T4 → T3conversion	Correlation between the G allele and rT3 levels	Interpreted as gain of function
Peeters et al. (31)	D2-ORFa-Gly3Asp −258 A/G rs12885300	Type 2 deiodinase (DIO2)	T4 → T3 conversion	Increased activity, changes in serum T3:FT4 ratio	
Medici et al. (33)	rs1382879 rs2046045 rs9687206 rs12515498 rs832790 rs1351283 rs989758	Phosphodiesterase 8B (PDE8B)	TSH signal transduction	Association with higher TSH levels	Medici et al. (33)
Medici et al. (33)	rs7714529	Phosphodiesterase 8B (PDE8B)	TSH signal transduction	Association with lower TSH levels	
Roef et al. (34)	rs5937843	Monocarboxylate transporter 8 (MCT8)	T3 cell membrane transporter	inverse association with FT4 concentrations	
Roef et al. (34)	rs6647476	Monocarboxylate transporter 8 (MCT8)	T3 cell membrane transporter	Inverse association with FT3 levels	
Carlé et al. (35)	rs17606253	Monocarboxylate transporter 10 (MCT10)	T3 cell membrane transporter	Carriers of both rs17606253 and rs225014 tend to prefer LT3:LT4 therapy	Synergistic effects with Thr92Ala variant

The discovery of the Thr92Ala polymorphism of the type 2 deiodinase gene (29), its association with subtle changes in thyroid hormone homeostasis (36), quality of life indices, and response to LT3:LT4 combination therapy (32) has prompted enthusiasm as a potential explanation of patient dissatisfaction (37). No study has prospectively analyzed the contribution of this polymorphism to quality of life or preference to LT3:LT4 combination therapy. Conversely, a prospective pharmacogenomic study carried out with healthy volunteers has demonstrated subtle differences in the pituitary thyroid axis response to TRH injection (38), in keeping with the hypothesis that the Ala 92 allele contributes to a decreased availability of T3 at the end organ tissues. The high prevalence (0.35) of the minor Ala92 allele across ethnicities (29) assures that ~50% of a random sample will be a carrier of this polymorphism in either homozygous or heterozygous. Thus, it is conceivable to design a study with selective recruitment based on the genotype, or alternatively allowing for a Mendelian randomization (i.e., enabling to perform a secondary analysis while knowing in advance the predicted allocation of the population). Although appealing, this strategy does not take in account other common polymorphisms in the deiodinase or other genes that could affect the thyroid hormone signaling. Indeed, we demonstrated a modulatory effect of the −258A/G D2, another common polymorphism of the type 2 deiodinase gene on the pituitary thyroid axis response to TRH injection (39). Paradoxically, this genetic variant (which is not associated with the Thr92Ala) is associated with increased sensitivity of the pituitary thyroid axis to the TRH stimulation. This finding suggests that the −258A/G D2 confers an increased deiodinase activity, likely exerted by removing a suppressor element in the promoter of the gene (40). Collectively, the data available indicate that the modulation of the thyroid axis, and by extension its response to exogenous replacement therapy, is exerted by a complex polygenic mechanism and no single genetic variant plays a prominent role. The effects of less common genetic variants may be clinically relevant when associated as haplotype in combination with other common variants, but designing a prospective study with such pharmacogenomic recruitment strategy would be extremely challenging. A more realistic strategy would be based on performing exploratory analyses after the completion of the data accrual.

### Serum T3 Levels

LT3:LT4 combination therapy or DTE administration should supply the exogenous T3 whose production is lost due to thyroid failure. Thus, targeting the recruitment to individuals with serum T3 levels at the low end or below normal limits is a viable possibility to increase the chances of detecting a statistical and clinically significant difference between treatment arms. This strategy has a clear theoretical appeal, and does not rely on assumptions about the relative role of specific genetic variants. On the other hand, there is no clear correlation between serum T3 levels and symptoms in hypothyroidism. Moreover, T3 levels are quite variable and exquisitely sensitive to changes in the health and nutrition status (41, 42). This could result in a major confounder in the selection of the study population. A corrective action to minimize this confounder would be to allow for a run-in period (with adequate replacement therapy and target TSH) aimed to confirm that the T3 levels are low independent from intervening pathologies. Additionally, individuals who have undergone weight loss, even remotely, should not be recruited because T3 levels remain low months and years following weight reduction (43). Similarly, chronic comorbidities or therapy with amiodarone, propranolol, or steroids can cause lowering of the serum T3 concentrations. While on the one hand, including these patients may tilt the study toward a more “real-life” effectiveness trial, on the other hand, these comorbidities and confounding factors may reduce the power of the study and make the interpretation of the findings challenging.

## Endpoints

The selection of study endpoint(s) is one of the most consequential decisions in the development of a successful clinical trial able to prove or disprove the efficacy and effectiveness of LT3:LT4 or DTE therapies. Just as in the selection of the study population, the choice of primary endpoint will be the result of a series of tradeoffs between scientific rigor, feasibility, and clinical relevance of the findings. In general terms, each clinical study stakes its endpoint “goalpost” to the measure that best reflects the efficacy of the drug tested. This is a well-established practice in cardiovascular medications, and more recently in glucose-lowering drugs studies where improvement in cardiovascular disease mortality is considered the ultimate primary outcome. Quite often, budgets and feasibility constrain limit the choice of primary endpoint to “second-best,” which is a valid proxy for the stated goals (e.g., composite cardiovascular endpoints). This approach is extremely complicated in the space of thyroid hormone replacement therapy where there is objective difficulty in determining the specificity of the symptoms, and the laboratory-based outcomes are affected by other biological factors. Below, we include some approaches to selecting the study endpoint that we believe are important in the decision-making process.

### Symptoms-Based Endpoints

The most common complaints attributed by patients to hypothyroidism concern quality of life. Fatigue, difficulties in concentrating, “mental fog,” and depression are the most common descriptors. Unfortunately, they are highly aspecific (28), affected by comorbidities and life stressors, and their quantification is challenging. Over the years, various general and thyroid disease-specific quality of life (QoL) instruments have been generated to interrogate the prevalence and to quantify the symptoms associated with thyroid disorders (3, 12, 44, 45). More recently, the ThyPRO has shown to be valid (46) and has been successfully used in large studies (47). Major advantages of disease-specific QoL instruments reside in the thorough validation process and in the possibility of deriving numerical scores that allow for detection of statistical differences among groups. The disadvantage of this approach resides in the variability of the symptoms among patients (28). In other words, while the plurality of patients complains of an aggregate of symptoms captured by the QoL instrument, at the individual level the symptom/sign that is debilitating may not have sufficient weight. Moreover, it is difficult to translate the clinical relevance of differences, albeit statistically significant ones, in QoL instruments to the day-to-day life experience of the individual patient. Therefore, the translation to clinical practice of the findings of a scientifically rigorous study could be challenging. A possible alternative strategy, not yet formally studied, would be to query the individual patient at the time of enrollment about the symptom which s/he finds more debilitating and attributes to hypothyroidism. Such symptoms (by default different among study participants) could be assessed on a visual analog scale, and then its differences could be evaluated during the study.

### Signs and Laboratory-Based Endpoints

Thyroid hormones affect virtually all organ systems, and there is a panoply of markers of thyroid hormone action which could be utilized as endpoints for a clinical trial. Body weight is probably the most widely recognized proxy for thyroid hormone action, even if the association between weight gain and hypothyroidism is at best tenuous. Importantly, weight gain is one of the most important drivers of dissatisfaction among patients affected by hypothyroidism (3, 4, 17, 45, 48) and conversely, weight loss was the greatest reason to declare satisfaction with the treatments among patients who preferred combination therapy or DTE on clinical trials (9, 10, 18, 22, 25). Moreover, a crossover study indicated that therapy with LT3 alone was associated with significant weight loss (49). Weight changes should thus be part of the measures captured in clinical trials, either as a secondary endpoint or as an explanatory variable for satisfaction. Extreme care should be taken in recording weight, including the use of standardized clothing and well-defined standard operating procedures to increase the reliability of the measurements. Depending on the study design, additional physiologic measurements may be considered depending on whether the study is more geared toward symptoms or exploration of the pathophysiology of the thyroid hormone replacement therapy. To this end, the measurement of energy expenditure by means of indirect calorimeter would provide important information (50). While hood calorimeters (metabolic carts) are commonly available, their sensitivity may not be sufficient to capture variations in energy expenditure, which are associated with small changes in body weight (49). Moreover, these systems do not capture all the components of energy expenditure. A more comprehensive strategy would be using extended recordings in whole room indirect calorimeters (51), but only few institutions have these instruments. One can envision, though, their use in proof of concept studies or in a nested study in the context of a large multicenter trial.

Lipid metabolism is directly affected by thyroid hormone action (52), and although extremely variable within the population, the intraindividual variation of serum lipids is remarkably small, making changes in serum lipids a reliable (and economical) variable which would be easy to capture. Although the cardiovascular system is an important target of thyroid hormone action (53, 54), a thorough assessment of the structural and functional changes would require dedicated resources beyond the scope of a clinical trial large enough to demonstrate effectiveness (i.e., patient-centered) results. It is conceivable nonetheless that proof of concept studies or nested studies within multicenter trials could address this topic. Although unlikely to show significant differences, blood pressure and heart rate should be recorded according to a well-defined standard operating procedure protocol to provide important information on potential safety signals. From a purely pathophysiologic exploration, additional targets of thyroid hormone action (e.g., body composition, sex hormone binding globulin, angiotensin converting enzyme, etc.) could be captured.

## Formulations and Dosing

The theory behind LT3:LT4 combination therapy or DTE administration is the replacement of endogenous T3 loss because of the development of hypothyroidism and/or due to a hypothetical deficit in peripheral conversion of exogenously-administered LT4 into T3 at the tissue target of the thyroid hormone action. Alternatively, one could aim to exploit the pharmacologic effects of T3 by providing higher doses than the estimated production from the thyroid. Irrespective, pharmacokinetics of LT3 and DTE, potential risks of overdosing, and feasibility are important determinants of the design of formulations and dosing frequency for a clinical trial.

The estimation of the contribution of the thyroid gland to the pool of circulating T3 suggests that the daily production of T3 is ~5 mcg/day, while the rest is the result of peripheral conversion of T4 (8). Thus, if the therapeutic goal is the replacement of this component, the formulations of LT3 in a LT3:LT4 combination should approximate this dose, ideally with adjustments for body weight, or limiting the recruitment to individuals within a specific range of body weight. Table 1 summarizes the treatment schemes in the LT3;LT4 combination therapy, and shows that with the exception of one trial in which the LT3:LT4 ratio was maintained at 1:14 (15), all the studies have used higher doses of LT3. In this series of articles, Drs. DiStefano and Jonklaas have provided an elegant theoretical modeling to titrate the LT3 dose in LT3:LT4 combination therapy schemes (55). Although appealing, this is not feasible in practice since the costs of individual formulations would be exorbitant. Indeed, in the USA, LT3 formulations are available in 5, 25, and 50 mcg strengths, so realistically the only options are fixed doses multiple of 2.5 mcg (half tablet) with matching placebo or for overencapsulation. Any greater degree of individualization would require a research pharmacy with the ability to produce tablets from bulk material. This is doable in small, proof of concept studies, but it would be highly impractical for large studies.

The pharmacokinetics (PK) characteristics of LT3 provide yet another layer of complexity since this formulation has a much shorter half-life when compared to LT4. Indeed, in our original crossover trial in which we treated hypothyroid patients devoid of endogenous production of thyroid hormone with LT3 or LT4 on a thrice daily administration scheme (56), we noted significant fluctuations of T3 while patients were treated with LT3 (49). Very recently, we formally characterized the PK of LT3 in the absence of endogenous or exogenous T4. The data are best explained by a two-compartment model with a fast distribution phase, and a much slower elimination phase with two distinct half-lives, 2.3 and 22.9 h, respectively (57). Based on these data, we were also able to generate a mathematical model able to predict the changes in T3 concentration and its fluctuations on various therapeutic schemes. Maintaining the same daily dose of LT3, the average serum concentration would not change between single vs. twice or administration regimens, but its variance between peak and trough would change dramatically. Moreover, we were able to predict the changes in T3 concentrations by adding LT3, either 3.75, 5, or 10 mcg twice daily, while decreasing the hypothetical LT4 dose of 112 mcg by 10, 25, or 50 mcg, respectively in a hypothetical 70 Kg patient. Our modeling indicates that in these scenarios, the mean serum T3 concentration would be increased from a baseline of 93 ng/dl (low end of normal range) by 26, 41, and 82 ng/dl, respectively. Of interest, only the highest LT3 dosing scheme would result in serum T3 concentrations above the upper level of reference, ~14% of the 24 h period (57). These theoretical data indicate that a twice daily administration scheme is feasible and would result in acceptable variations of serum T3 concentrations. On the other hand, such a scheme would still require a twice daily administration regimen, which is not ideal for a lifelong treatment.

There is general consensus that peaks of serum T3 concentrations may result in acute and potentially lethal toxicity, particularly at the level of the cardiovascular system which would be directly exposed to rapid rise in T3 should the administration be performed on a single daily regimen. This concern is supported by the well-known toxic effects of sustained thyrotoxicosis and, to some degree, by the demonstration of rapid, non-genomic effects of thyroid hormone which are exerted at the level of the vascular endothelium (58, 59). Conversely, it is worth nothing that while the non-genomic effects of thyroid hormone are well-characterized *in vitro*, their clinical relevance is not clear. Of interest, a PK study performed in healthy volunteers with pharmacologic doses of LT3 did not show any measurable change in blood pressure until 5 h following the drug administration (60), indicating that the action of thyroid hormone on the cardiovascular system (limited to blood pressure and heart rate) is mediated by its effect on gene transcription rather than rapid, non-genomic mechanisms. Whether thyroid hormones exert other less evident but potentially clinically relevant non-genomic effects on the cardiovascular system should be explored in detailed studies. This is important because the scientific community would not accept a single daily administration regimen of LT3 (with consequent peaks and throughs) in the absence of a clear demonstration of the safety of this scheme.

## Study Design

The duration of the study and its design are critical decision points in the development of an adequately powered and internally valid study to prove or disprove the efficacy of LT3:LT4 combination therapy or DTE for the treatment of hypothyroidism. There is clear consensus about the need to perform double blind intervention with an adequate observation period to mitigate placebo effect and to allow for the effects of different formulations of thyroid hormone to exert their actions on the various endpoints, be they anthropometrics, laboratory-based, or QoL (2). Additionally, repeated measures can demonstrate trends or regression to the mean of various endpoints. Considering that thyroid hormone replacement is a lifelong therapy, and that (relative to LT4) a steady state is reached after 6 weeks from the dose adjustment, a long observation period (12 or 24 months) would be ideal. The long duration of the study would also allow for therapy adjustments, and also allow for gathering data on potential toxicity. Conversely, since the study drugs are available in commerce, one can expect a significant attrition during the study, with participants who do not experience the expected improvement being likely to drop out and to request their physicians to prescribe LT3:LT4 combination therapy or DTE. A crossover design is appealing because of the increase in statistical power due to the ability of performing paired analyses (25, 49). This advantage is mitigated by the potential carryover effect of the first study drug on the second treatment, and the risk of loss of data in case of drop out.

## Designing the “Ideal Study”

**Figure 1:**
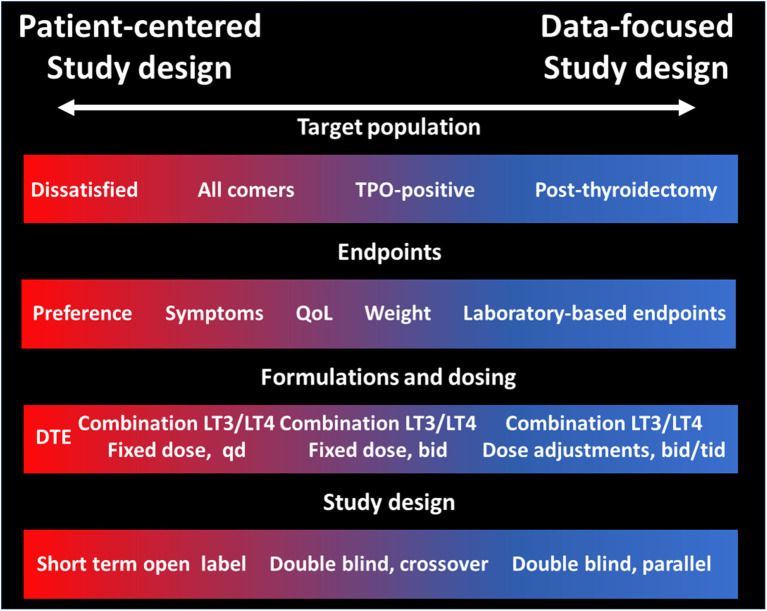
Tradeoffs in study design of LT3:LT4 combination therapy or DTE vs. LT4 alone for the treatment of hypothyroidism. Tradeoffs in four domains of a clinical trial directed to assess the efficacy and effectiveness of therapies other than LT4 alone vs. LT4. The left part of the panels (red) indicates choices more suitable for a patient-centered study, while the right side of the panels indicates choices more suitable for a data-focused study.

The lack of definitive data on the efficacy and effectiveness of LT3:LT4 combination (or DTE) for the treatment of hypothyroidism has preempted the professional societies to endorse these therapeutic modalities. This is completely understandable due to the potential for toxicity due to excessive LT3 dosing, and the increased costs and complexities in the treatment schemes when compared to once-a day LT4. There is obvious need of compelling data that could prove or disprove the value of therapies other than LT4 alone. To this end, the stakes in designing such a study are enormous, and the tradeoffs described in the previous sections need to be carefully considered in order to make conscious decisions rather than being forced to compromise to a study that may not have sufficient statistical power or have a primary endpoint that does not sufficiently address the study question. The major decision points researchers (and funding agencies) will need to face (Figure 1) are about the primary endpoint and on the dosing and ratio LT3:LT4 formulation or accepting the non-physiologic T3:T4 content of DTE. While there are many validated QoL, it may be challenging correlating the changes in these instruments with the symptoms of the individual patient. Similarly, while changes in laboratory-measured parameters are easily quantifiable, their clinical significance and their relevance to the “dissatisfaction” question could be disputed. Relative to the study drug formulation, the major decision point is whether to aim for a “physiologic” replacement of the T3 lost from lack of thyroid function or to use higher doses to exploit the pharmacologic effects of T3, possibly raising the serum concentrations above the individual's levels before developing hypothyroidism. This latter approach may provide a greater effect size, but conversely could expose patients to untoward toxicity. Likewise, a better understanding of the clinical relevance (or lack thereof) of rapid, non-genomic effects of T3 could provide the rationale to designing once daily administration schemes which in turn could improve the adherence to the regimen. Small, proof of concept preliminary studies could provide empirical information, avoiding the scientific risk of relying on theoretical modeling, convenience, or experts' opinions.

## Conclusions

Dissatisfaction toward LT4-only therapy among patients affected by hypothyroidism is a common and well-recognized problem. While all the stakeholders (patients, pharmaceutical companies, physicians, professional organizations, and funding agencies) recognize the need of evidence generated from well-conducted studies, there is still lack of consensus about how an “ideal study” would be structured. Clinical investigators should approach the various tradeoffs in the study design with a clear understanding of how these decisions will affect the internal validity of the study, as well as the applicability and relevance of the findings to scientific community and to the patients, who ultimately are the most important stakeholders.

## Author Contributions

RM reviewed the literature and contributed to the initial manuscript draft. FC designed the manuscript scope, structure, take home message, and wrote the final version of the manuscript. All authors contributed to the article and approved the submitted version.

## Conflict of Interest

The authors declare that the research was conducted in the absence of any commercial or financial relationships that could be construed as a potential conflict of interest.
